# Child‐To‐Adult Body Size Change and Risk of Depression and Anxiety: A Large Prospective Cohort Study

**DOI:** 10.1155/da/6154705

**Published:** 2026-09-03

**Authors:** Ke Peng, Jian Peng, Minzhi Xu, Yun Liu, Yanhong Gong

**Affiliations:** ^1^ Department of Social Medicine and Health Management, School of Public Health, Tongji Medical College, Huazhong University of Science and Technology, Wuhan, Hubei, China, hust.edu.cn; ^2^ Department of ENT, Tongji Hospital, Tongji Medical College, Huazhong University of Science and Technology, Wuhan, Hubei, China, hust.edu.cn

**Keywords:** anxiety, body size trajectories, depression, life-course epidemiology, mental health inequalities

## Abstract

**Background:**

Body size across the life course has been associated with long‐term mental health outcomes, yet most previous studies have examined body size at a single developmental stage. Whether changes in relative body size from childhood to adulthood represent a distinct life‐course marker of vulnerability to later depression and anxiety remains insufficiently understood. We examined the associations between child‐to‐adult body size changes and the risk of depression and anxiety and explored whether lifestyle factors and chronic diseases partly explained these associations.

**Methods:**

This study included 447,821 participants from the UK Biobank. Childhood body size at age 10 years was retrospectively self‐reported as relative body size, and adult body size was assessed using measured body mass index (BMI) at baseline. Depression and anxiety were identified from electronic health records questionnaires, including the Patient Health Questionnaire‐9 and the Generalized Anxiety Disorder‐7. Cox proportional hazards models were used to estimate associations with incident diagnoses, and mixed‐effects linear regression models were used to examine symptom severity scores. Exploratory mediation analyses were conducted to assess the extent to which lifestyle factors and chronic diseases, including type 2 diabetes (T2D), hypertension and cardiovascular disease (CVD), statistically accounted for the observed associations.

**Results:**

In the fully adjusted models, child_Low_–adult_High_ group had the highest risk of major depression (HR: 1.90, 95% CI: 1.79−2.01) and anxiety (HR: 1.58, 95% CI: 1.48−1.68) compared with the child_Average_–adult_Average_ group. Unhealthy lifestyle factors and chronic diseases together statistically accounted for 21.9% of the association between the child_Low_–adult_High_ group and major depression and 22.2% of the association between the child_Low_–adult_High_ group and anxiety.

**Conclusions:**

Distinct body size changes from childhood to adulthood are associated with differential risks of depression and anxiety. Individuals transitioning from childhood leanness to adult heaviness may represent a particularly vulnerable life‐course group. These findings support the importance of life‐course approaches to understanding mental health vulnerability and identifying opportunities for early prevention.

**Summary**

• Childhood leanness was identified as an overlooked predictor of adult depression and anxiety.
• The child_Low_–adult_High_ changes showed the greatest risk for mental health outcomes.
• Lifestyle factors and chronic diseases jointly mediated ~22% of the associations.
• Findings underscore the importance of life‐course weight management for mental health promotion.

## 1. Introduction

Depression and anxiety are among the most prevalent and disabling forms of psychopathology worldwide, yet their developmental origins remain incompletely understood ([1, 2], pp. 1980–2013). Globally, nearly one in seven people was living with a mental disorder in 2021, with anxiety and depressive disorders being among the most common conditions. These conditions are associated with substantial distress, functional impairment and increased suicide risk, underscoring the need to identify life‐course markers of mental health vulnerability. In the United Kingdom, mental health has also become a major public health concern. Recent national data from England showed that one in five adults had a common mental health condition in 2023/2024, with generalised anxiety disorder and depressive episode among the most prevalent conditions. The prevalence of common mental health conditions among adults aged 16–64 years increased from 18.9% in 2014 to 22.6% in 2024 [3]. These figures highlight the value of using large UK‐based cohort data to examine potentially modifiable factors associated with later depression and anxiety.

A growing body of research suggests that risk for common mental disorders may emerge from exposures that accumulate across the life course rather than from isolated factors occurring at a single developmental stage [4]. Body size is one such exposure because it is socially, behavioural and metabolically patterned across the life course. Obesity has been described as a global epidemic in recent years [5], with more than 650 million adults worldwide living with obesity [6]. While obesity is often associated with physical health, mental disorders due to weight‐based bullying, discrimination, low self‐esteem, metabolic disorders and functional impairment are also of increasing concern [7, 8]. Emerging evidence has suggested that obese adults are at higher risk for depression [9]. Longitudinal studies of young people have also found that high body mass index (BMI) during adolescence is significantly associated with adverse mental health later in life [10]. However, current studies have focused on the association between body size at a single point in time (such as adolescence or middle age) and mental health, and little attention has been paid to the relationship between child‐to‐adult body size change and subsequent mental health. Cumulative advantage (disadvantage) theory and life‐course theory emphasise that early adverse circumstances may accumulate over time, creating heterogeneity in individual health and contributing to health inequalities [4, 11]. Understanding the association between long‐term body shape changes from childhood to adulthood and mental health from a life‐course perspective may further guide and improve weight‐related health strategies and practices to promote public mental health. However, empirical evidence examining the joint influence of childhood and adult body size on mental health remains limited.

Moreover, the mechanisms underlying these associations are not well understood. Lifestyle behaviours are one plausible domain through which child‐to‐adult body size change may be linked to later depression and anxiety. Upward shifts in relative body size from childhood to adulthood may be accompanied by behavioural patterns such as physical inactivity, an unhealthy diet, prolonged sedentary behaviour, short or long sleep duration, smoking or frequent alcohol consumption. These behaviours may contribute to mental health risk through several mechanisms, including poorer sleep quality, inflammation, metabolic dysregulation, reduced social participation and lower perceived control over health. Obesity‐related chronic diseases represent another plausible explanatory domain. Type 2 diabetes (T2D), hypertension and cardiovascular disease (CVD) were common cardiometabolic conditions closely related to a higher adult body size and weight gain. These conditions may increase psychological distress through disease burden, treatment demands, functional limitation, vascular and inflammatory processes and concerns about future health.

However, empirical evidence examining child‐to‐adult body size change in relation to later depression and anxiety remains limited, and the extent to which lifestyle behaviours and cardiometabolic diseases explain these associations is not well understood. To address these gaps, the present study used data from the UK Biobank to examine the associations between child‐to‐adult body size changes and subsequent risks of depression and anxiety. We further explored whether lifestyle factors and chronic diseases may explain these associations. We hypothesised that (1) body size change characterised by adult heavy weight would be associated with higher risks of depression and anxiety, and (2) these associations would be partially mediated by unhealthy lifestyle behaviours and obesity‐related chronic diseases (T2D, hypertension and CVD).

## 2. Methods

### 2.1. Study Design and Participants

The present analysis was based on data from the UK Biobank, a large‐scale prospective cohort that enrolled over 500,000 adults across the United Kingdom between 2006 and 2010. Participants visited one of 22 dedicated assessment centres where physical measurements were taken and detailed questionnaires on health and lifestyle were completed. Information on mental health was later collected through a web‐based follow‐up questionnaire administered in 2016–2017 [12]. Detailed descriptions of the cohort design and data collection procedures have been published elsewhere [13, 14]. For the current study, we excluded individuals who were lost during follow‐up, reported a prior diagnosis of major depression or anxiety disorders, were pregnant at baseline, or had missing information on childhood or adult body size as well as relevant covariates Because body size distributions vary across ethnic groups, analyses were restricted to participants of European ancestry to reduce potential heterogeneity. After applying these criteria, 447,821 individuals remained in the analytic sample. Among them, 143,147 participants completed the mental health follow‐up survey in 2016–2017 and were included in the prospective analyses examining the association between trajectories of body size from childhood to adulthood and subsequent symptoms of depression and anxiety. The participant selection process is illustrated in Figure S1.

The UK Biobank received ethical approval from the North West Multi‐Centre Research Ethics Committee. All participants provided electronic informed consent at the baseline assessment. The present analyses were conducted under the approved Application Number 88159.

### 2.2. Exposure Assessment

Body size information during childhood was obtained through the touchscreen questionnaire: ’When you were 10 years old, compared to average, would you describe yourself as: plumper, about average, or thinner?’. Three recalled relative childhood body size groups were defined by the options ’plumper’(child_High_), ’about average’ (child_Average_) and ’thinner’ (child_Low_), accounting for 15.9%, 51.2% and 32.8% of the study population, respectively. This measure should be interpreted as recalled relative childhood body size rather than objectively measured childhood BMI. Prior studies examining polygenic scores against measured BMI in large cohorts support the validity of this UK Biobank childhood body size indicator [15, 16], although recall error and misclassification cannot be fully excluded.

Adult anthropometric measurements were obtained at baseline by trained research staff. The participants were measured without shoes and while wearing light clothing. Standing height was assessed in the barefoot position using a Seca 202 stadiometer (Birmingham, UK), and body weight was recorded using a Tanita BC‐418MA body composition analyser (Amsterdam, Netherlands). The BMI was derived as body weight in kilograms divided by height in metres squared.

**Figure 1:**
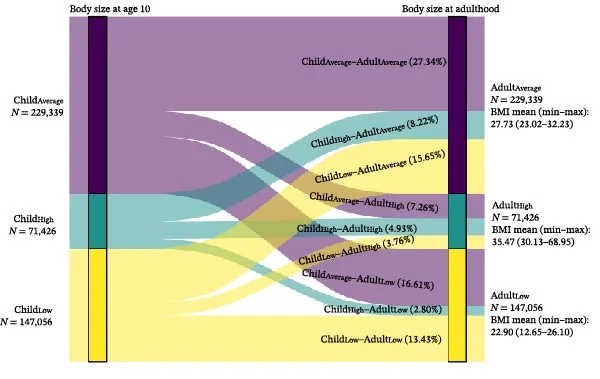
Definition and proportions for body size change from childhood to adulthood.

Initially, adult body size categories were considered according to the WHO classification thresholds for underweight, normal weight, overweight and obesity. However, these clinical BMI categories were not directly aligned with the recalled childhood question, which assessed body size relative to peers rather than clinical weight status. In addition, the number of individuals classified as underweight was very small (*N* = 2244), accounting for only 0.5% of the sample [17]. Moreover, a large proportion of participants (66.6%; *N* = 298,223) fell into the overweight or obese categories. Applying WHO thresholds to define child‐to‐adult body size change groups therefore resulted in highly imbalanced groups in terms of both sample size and disease occurrence. To construct relative adult body size groups that were more comparable with the relative childhood body size question, adult body size was characterised using BMI residuals derived from a regression model that incorporated age, age squared and sex as predictors (BMI ~ age + age^2^ + sex) (Figure S2). Based on the distribution of these residuals, three adult body size categories were generated so that their proportions corresponded to those observed for childhood body size: adult_High_ (15.9%), adult_Average_ (51.2%) and adult_Low_ (32.8%). This residual‐based classification was not intended to replace clinical BMI categories; rather, it was used to create relative adult body size categories for life‐course change analysis. Similar approaches have been used in previous studies of child‐to‐adult body size change ([18], p. 2; [16]). Detailed information about body size changes from children to adults is shown in Figure 1, and information about body size changes stratified by sex is shown in Figure S3.

### 2.3. Outcome Assessment

Consistent with previous studies, incident hospital‐record diagnoses of major depression or anxiety were identified when a physician documented either a primary or secondary diagnosis coded according to the International Classification of Diseases, 10th Revision [19, 20]. Major depressive disorder was defined using the first recorded diagnosis with codes F32 or F33, whereas anxiety disorders were identified using codes F40–F43. (Table S1). For each outcome, follow‐up time was calculated from baseline until the earliest of the following events: the first recorded diagnosis of the disorder of interest, death or the administrative end of follow‐up (30 September 2021). Depression and anxiety outcomes were analysed independently, meaning that the occurrence of one condition did not censor follow‐up for the other. Hospital records are likely to capture clinically recognised diagnoses and may under‐ascertain milder or untreated symptoms.

To further examine whether reliance on hospital records might have influenced the observed associations, additional analyses were conducted among participants who completed the mental health follow‐up questionnaire. In this subsample, symptoms of depression and anxiety were assessed during the follow‐up survey using the Patient Health Questionnaire‐9 (PHQ‐9) and Generalized Anxiety Disorder‐7 (GAD‐7), respectively [21, 22]. The PHQ‐9 includes nine items scored from 0 to 3, yielding a total score ranging from 0 to 27, while the GAD‐7 includes seven items scored from 0 to 3, yielding a total score ranging from 0 to 21. Higher scores indicate more severe symptoms. In the present analytic subsample, the internal consistency was good, with a Cronbach’s alpha of 0.842 for PHQ‐9 and 0.898 for GAD‐7 in Table S6. These questionnaire‐based analyses were interpreted as complementary evidence on symptom severity rather than as directly equivalent to hospital‐record diagnoses.

### 2.4. Potential Mediators and Covariates

A comprehensive lifestyle index was developed to capture overall health‐related behaviours. The index incorporated six behavioural domains frequently used in prior epidemiological studies: smoking, alcohol intake, physical activity, sleep duration, sedentary behaviour and habitual diet [20, 23]. For each domain, participants were classified as having either a healthy or an unhealthy behaviour according to previously used criteria. Current smoking was classified as unhealthy, whereas non‐current smoking was classified as healthy. Alcohol intake was classified as unhealthy if participants reported drinking alcohol daily or almost daily. Physical activity was quantified according to the International Physical Activity Questionnaire scoring guidelines and expressed in metabolic equivalent units; insufficient physical activity was classified as unhealthy according to recommended thresholds [24]. Sleep duration was derived from self‐reported hours of sleep and classified as unhealthy when it fell outside the recommended range [25]. Sedentary behaviour was assessed using television viewing time, with prolonged television viewing classified as unhealthy according to previously used thresholds. Diet quality was evaluated using a score constructed from national dietary recommendations and previously validated criteria. Seven food components were included: fruit intake, vegetables, whole grains, oily fish, refined grains, processed meat and red meat consumption [20, 26]. Participants meeting at least five of these recommended dietary targets were classified as having a healthy diet. Each unhealthy lifestyle behaviour was assigned one point, resulting in an unhealthy lifestyle index ranging from 0 to 6, with higher scores indicating a greater accumulation of unhealthy behaviours. Detailed definitions of healthy and unhealthy categories for each lifestyle domain are provided in Table S2. Each of the lifestyle factors had a missing proportion of less than 1%, except for physical activity (18.25%) and diet (22.37%). Missing observations were handled using multiple imputation by chained equations with five imputed datasets.

T2D was defined using the case identification algorithm developed for the UK Biobank dataset [27]. CVD and hypertension were identified through linked hospital records using diagnostic codes from the International Classification of Diseases (9th and 10th revisions), supplemented with self‐reported information on physician diagnoses and relevant medical procedures [28]. Details about the definitions of potential mediators are described in Supporting Methods.

Potential confounding variables included age, sex, educational attainment, the Townsend deprivation index and assessment centres. These covariates were selected based on previous literature on body size and mental health and on their availability in the UK Biobank, with the aim of accounting for demographic, socio‐economic and recruitment‐site differences that could be associated with both body size and mental health outcomes. Age and sex were included because they are strongly related to the body size distribution and risks of depression and anxiety. Educational attainment and the Townsend deprivation index were included to capture individual‐ and area‐level socio‐economic conditions that may influence both body size development and mental health. An assessment centre was included to account for recruitment‐site differences and potential geographic variation in data collection. The educational attainment was categorised according to the UK education system: degree level, A‐level or equivalent, O‐level/GCSE or equivalent, vocational qualifications, other professional qualifications or none. The socio‐economic status was represented by the Townsend deprivation index, which is derived from national census data, with higher values indicating greater socio‐economic disadvantage [20].

### 2.5. Statistical Analysis

Participant characteristics at baseline were summarised across the nine life‐course body size categories using means and standard deviations for continuous variables and frequencies and percentages for categorical variables.

Associations between child‐to‐adult body size changes and the incidence of major depression and anxiety were evaluated using Cox proportional hazard regression models. The reference category used was the child_Average_–adult_Average_ group. Among participants who completed the mental health follow‐up survey, additional analyses examined the relationship between body size trajectories and symptom severity scores derived from the PHQ‐9 and GAD‐7. These associations were analysed using mixed‐effects linear regression models. The centre was incorporated as a random intercept to account for potential clustering and residual variation related to examination sites. All models included the covariates described above.

To explore whether lifestyle behaviours and chronic conditions (T2D, hypertension and CVD) statistically accounted for the associations between child‐to‐adult body size change patterns and mental health outcomes, exploratory mediation analyses were conducted [29]. These analyses were used to estimate the total, direct and indirect effects within a counterfactual framework. Because adult body size, lifestyle behaviours and chronic conditions were assessed at the baseline examination, the mediation analyses were interpreted as exploratory and should not be considered evidence of definitive causal pathways. Mediation and outcome models were fitted simultaneously, adjusting for all confounders as well as the remaining explanatory factors. Confidence intervals for the proportion accounted for were derived from 1000 quasi‐Bayesian Monte Carlo simulations.

Missing data were handled using multiple imputation by chained equations under the missing‐at‐random assumption. The imputation model included the exposure, outcomes, potential explanatory factors, covariates and follow‐up information used in the analytic models. Five imputed datasets were generated, and estimates were combined using Rubin’s rules. Because physical activity and diet had relatively higher proportions of missingness than other lifestyle variables, we conducted complete‐case analyses among participants with complete lifestyle information to assess the robustness of the exploratory mediation results to missing data handling.

We performed several additional analyses. First, the associations between body size and mental health outcomes were examined separately for childhood and adult body sizes. Second, we stratified the analyses by sex to explore potential differences in associations between child‐to‐adult body size changes and mental health outcomes in men and women. Third, to minimise potential influences of the COVID‐19 pandemic on outcome ascertainment, the follow‐up time was truncated at 31 December 2019. Finally, exploratory mediation analyses were repeated among participants with complete lifestyle information to evaluate the robustness of the findings to the missing data handling.

All statistical analyses were carried out using R Version 4.0.5. Statistical significance was defined using two‐sided tests with a threshold of *p*  < 0.05.

## 3. Results

### 3.1. Baseline Characteristics

Among the 447,821 participants (mean age 56.73 ± 8.02 years old), 203,442 (45.4%) were male, and 244,379 (54.6%) were female. During a median follow‐up of 12.56 years, 22,396 participants (5.0%) developed depression and 19,142 (4.3%) developed anxiety. 45.70% of participants maintained the same body size category from childhood to adulthood, while 27.63% went from a lower body size category in childhood (child_Low_/child_Average_) to a higher body size category in adulthood (adult_Average_/adult_High_) and 26.67% moved from a higher body size category in childhood (child_High_/child_Average_) to a lower body size category in adulthood (adult_Average_ or adult_Low_). Baseline characteristics of the nine child‐to‐adult body size groups are reported in Table S3. Compared with excluded participants, included participants were slightly older, more socio‐economically advantaged and less likely to have T2D, hypertension or CVD and all were of White ethnicity by design after the analytic restriction (Table S4).

### 3.2. Child‑to‑Adult Body Size Change and Risk of Depression and Anxiety

**Figure 2:**
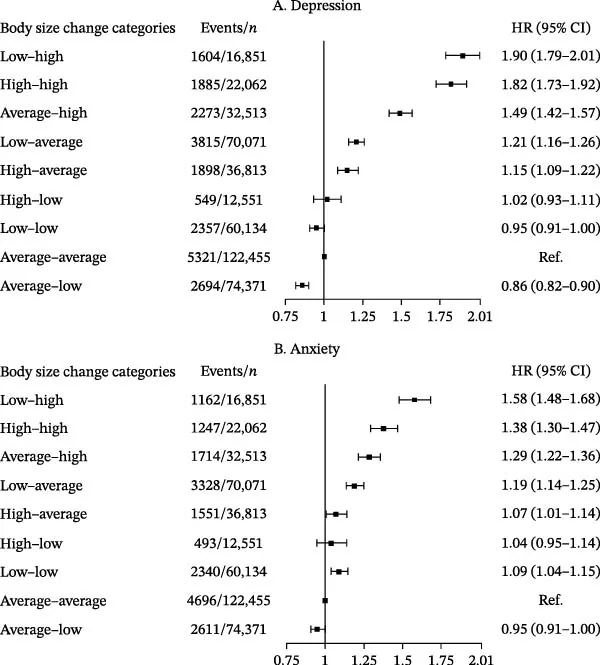
Hazard ratios and 95% CIs for major depression (A) and anxiety (B) associated with different child–adult body size categories. The model was adjusted for age, sex, educational attainment, Townsend deprivation index and assessment centres.

**Figure 3:**
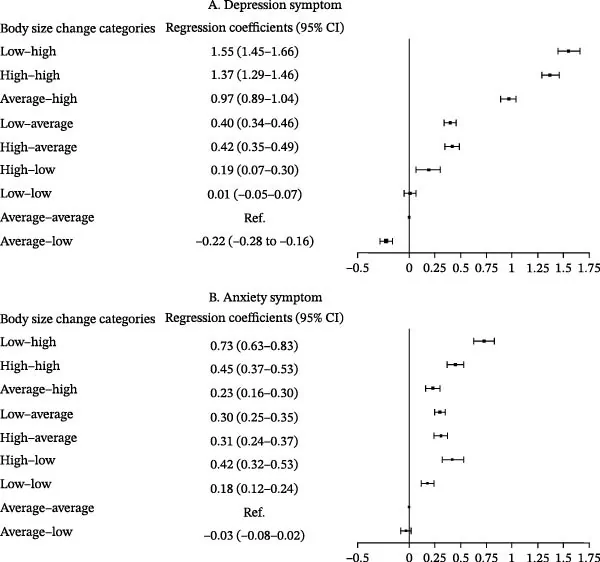
Differences in PHQ‐9 (A) and GAD‐7 (B) scores among the nine child–adult body size groups. The model was adjusted for age, sex, educational attainment and Townsend deprivation index. The assessment centre was additionally controlled as a random effect.

Figure 2 showed the associations of child‑to‑adult body size change with major depression and anxiety. Of the nine child–adult body size groups, the three adult_High_ groups were associated with the highest incidence of major depression and anxiety. In the fully adjusted models, the child_Low_–adult_High_ group had the highest risk of major depression (HR: 1.90, 95% CI: 1.79–2.01) and anxiety (HR: 1.58, 95% CI: 1.48–1.68) compared with the child_Average_–adult_Average_ group. The estimates were higher than those observed for participants who had high or average body size in childhood and high body size in adulthood (HR_depression_ for child_High_–adult_High_ group: 1.82, 95% CI: 1.73–1.92; HR_depression_ for child_Average_–adult_High_ group: 1.49, 95% CI: 1.42–1.57; HR_anxiety_ for child_High_–adult_High_ group: 1.38, 95% CI: 1.30–1.47; HR_anxiety_ for child_Average_–adult_High_ group: 1.29, 95% CI: 1.22–1.36). In addition, we also found that the child_Average_–adult_Low_ group had the lowest risk of major depression and anxiety. Similar patterns were observed in associations with PHQ‐9 and GAD‐7 symptom scores (Figure 3).

### 3.3. Exploratory Mediation Effects

**Figure 4:**
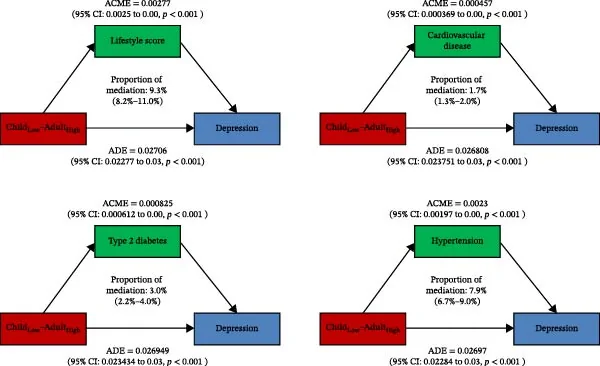
Exploratory contribution of common chronic diseases (including T2D, hypertension and CVD) and lifestyle factors to the association between the child_Low_–adult_High_ group and depression. ADE, average direct effects; ACME, average mediation effects.

**Figure 5:**
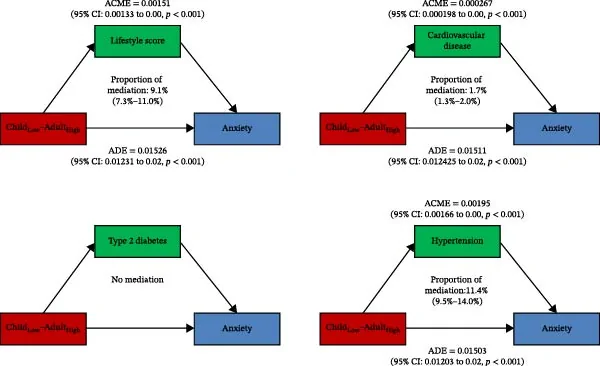
Exploratory contribution of common chronic diseases (including T2D, hypertension and CVD) and lifestyle factors to the association between the child_Low_–adult_High_ group and anxiety. ADE, average direct effects; ACME, average mediation effects.

Before conducting exploratory mediation analyses, we first examined the correlations between the child_Low_–adult_High_ contrast and each potential explanatory factor and between these factors and depression or anxiety (Table S5). These findings supported the use of these variables as potential explanatory factors in exploratory mediation analyses. In exploratory mediation analyses, unhealthy lifestyle factors (9.3%), CVD (1.7%), T2D (3.0%) and hypertension (7.9%) together statistically accounted for 21.9% of the association between the child_Low_–adult_High_ and major depression. Similarly, unhealthy lifestyles (9.1%), CVD (1.7%) and hypertension (11.4%) together accounted for 22.2% of the association between the child_Low_–adult_High_ and anxiety. These estimates suggest that the measured lifestyle and cardiometabolic factors explained part, but not all, of the observed associations. The total, indirect and direct effect estimates are shown in Figures 4 and 5. Detailed estimates of the indirect, direct and total effects, together with model R2 and AIC values, are shown in Table S7.

### 3.4. Additional Analyses

Additional analyses examining childhood and adult body size separately, sex‐stratified associations, censoring before the COVID‐19 pandemic and complete‐case exploratory mediation analyses produced broadly similar patterns (Figures S4–S7). These analyses supported the robustness of the main associations, although they do not eliminate the possibility of residual confounding or selection bias.

## 4. Discussion

In this large prospective analysis of UK Biobank participants, child‐to‐adult body size changes were associated with subsequent risks of depression and anxiety. Participants who reported lower relative body size in childhood and had a higher relative body size in adulthood showed the highest risks of both outcomes. Similar patterns were observed for hospital‐record diagnoses and for questionnaire‐based symptom scores, suggesting that the findings were not limited to one type of mental health assessment. Exploratory mediation analyses indicated that unhealthy lifestyle factors and obesity‐related chronic diseases statistically accounted for approximately one‐fifth of these associations.

Our findings extend previous research that has mainly examined the body size at a single developmental stage. Prior studies have linked childhood, adolescent or adult excess body weight with later depressive symptoms and other adverse mental health outcomes. For example, a longitudinal investigation in a Mediterranean population reported that women with elevated body weight in early childhood and marked weight gain during young adulthood were more likely to develop depressive symptoms later in life [30]. Evidence from the Northern Finland Birth Cohort similarly suggested that obesity during adolescence is linked to an increased likelihood of depression in adulthood, with adolescent and adult overweight representing an important risk factor for depressive disorders among women [31]. Compared with these studies, our analysis focused on relative body size changes between childhood and adulthood rather than body size at only one life stage. This distinction is important because child‐to‐adult body size change may capture accumulated psychosocial, behavioural and metabolic vulnerability across developmental stages.

Our study indicates that relatively low body size during childhood may also be associated with poorer mental health in adulthood; particularly, those who moved from a lean body size in childhood to a higher body size in adulthood appeared to face the greatest risk of both depression and anxiety. This finding emphasised the importance of maintaining healthy body weight trajectories throughout the life course. From a life‐course perspective, the particularly elevated mental health risks observed among individuals transitioning from childhood leanness to adult obesity may reflect an accumulation of social and psychological vulnerabilities over time. Early‐life body size can shape self‐perception, social experiences and expectations regarding health and body image, while subsequent weight gain in adulthood may introduce additional psychosocial stressors, including weight‐related stigma, social comparison and perceived loss of bodily control [18]. The mismatch between early‐life body size and later‐life body status may therefore represent a critical change in which cumulative exposures across developmental stages interact to influence mental health vulnerability.

Exploratory sex‐stratified analyses showed broadly similar patterns in men and women: the child_Low_–adult_High_ group generally had the highest risks of depression and anxiety in both sexes. The only notable exception was depression among women, for which the child_High_–adult_High_ and child_Low_–adult_High_ groups showed comparable risk estimates. This suggests that both persistently higher relative body size and upward change to a higher adult body size may be relevant to depression risk among women. This interpretation is consistent with previous evidence suggesting that body weight, body dissatisfaction and weight‐related social experiences may be particularly salient for women’s mental health.

The exploratory mediation findings provide some insight into, but do not fully explain, the elevated risks observed in the child_Low_–adult_High_ group. In line with the life‐course interpretation above, a substantial upward shift in relative body size may be accompanied by behavioural and cardiometabolic changes that are themselves relevant to mental health. Unhealthy lifestyle behaviours, including poor diet, physical inactivity, inadequate sleep, smoking, frequent alcohol use and sedentary behaviour, may reflect modifiable behavioural correlates of this body size change pattern [23, 25]. Cardiometabolic conditions may also contribute to later psychological vulnerability through disease burden, functional limitation, treatment demands and concerns about future health [18]. In our exploratory mediation analyses, lifestyle factors, hypertension, CVD and T2D together statistically accounted for approximately one fifth of the associations with depression and anxiety. Thus, these factors should be interpreted as partial explanatory markers rather than as evidence of a complete or causal mechanism. The remaining association may reflect other unmeasured life‐course processes, including early socio‐economic conditions, childhood nutrition, parental influences, childhood mental health, genetic susceptibility, body dissatisfaction, weight stigma, social adversity and healthcare‐seeking behaviour.

This investigation represents one of the largest longitudinal analyses examining child‐to‐adult body size changes in relation to the subsequent risks of depression and anxiety. The study included more than 400,000 participants and a mean follow‐up period of ~12.6 years, which generated a substantial number of outcome events and provided sufficient statistical power to evaluate these associations. The use of both hospital‐record diagnoses and questionnaire‐based symptom scores also allowed us to examine whether the observed associations were broadly consistent across different mental health assessments. Nevertheless, several limitations should be considered.

First, UK Biobank participants are not representative of the general population and tend to be healthier and more socio‐economically advantaged than the wider UK population. Questionnaire‐based analyses were further restricted to participants who completed the mental health follow‐up survey. Therefore, the absolute risks observed in this study should not be interpreted as population prevalence estimates. Second, analyses were restricted to participants of European ancestry to reduce heterogeneity in body size distributions, and caution is warranted when extrapolating the findings to other ethnic, social and cultural contexts.

Third, childhood body size was based on a retrospective self‐report at age 10 and captured perceived body size relative to peers rather than objectively measured childhood BMI. This may introduce recall error and misclassification, and participants’ current body size or health status may have influenced their recall. Although previous validation studies support the usefulness of this UK Biobank measure as an indicator of early‐life adiposity, the possibility of a biased recall cannot be excluded [15, 16]. Accordingly, our findings should be interpreted as associations involving recalled relative childhood body size rather than objectively measured childhood adiposity. Fourth, adult body size was classified using age‐ and sex‐adjusted BMI residuals to create relative categories comparable with the childhood body size question. This approach improves comparability across life stages but is less clinically intuitive than the standard BMI categories. Adult_High_, adult_Average_ and adult_Low_ should be interpreted as relative adult body size groups rather than clinical obesity, normal weight or underweight categories.

Fifth, although participants with diagnosed depression or anxiety at baseline were excluded, undiagnosed or subclinical symptoms may have been present before baseline and could have influenced adult body size, lifestyle behaviours, healthcare use or recall of childhood body size. Therefore, reverse causality cannot be fully ruled out, and the findings should be interpreted as prospective associations rather than evidence of a strictly unidirectional causal relationship. Sixth, adult body size, lifestyle behaviours and chronic diseases were assessed at baseline. Therefore, the temporal ordering required for strong causal mediation claims could not be fully established. Finally, residual confounding cannot be excluded because genetic predisposition, early‐life socio‐economic conditions, childhood dietary patterns, parental influences and childhood mental health were not fully captured in the available data.

In conclusion, individuals who experienced a change from relatively low body size during childhood to higher body size in adulthood exhibited the greatest risk of developing depression and anxiety, partly mediated through unhealthy lifestyles and chronic diseases (T2D, hypertension and CVD). Our study revealed the importance of promoting healthy weight management throughout the life course and highlighted the need for future studies with repeated measurements from childhood to adulthood to clarify causal mechanisms and inform prevention strategies.

## Author Contributions

**Ke Peng and Jian Peng**: methodology, data analysis, writing – original draft preparation, revision of the manuscript. **Minzhi Xu**: conceptualisation, methodology, validation. **Yanhong Gong and Yun Liu:** supervision, visualisation, administrative support, critical revision of the manuscript.

## Funding

The authors have nothing to report.

## Disclosure

The study was not pre‐registered. The analyses were conducted using existing observational data from the UK Biobank, and the research questions and analytical strategy were developed as part of the secondary data analysis.

## Conflicts of Interest

The authors declare no conflicts of interest.

## Supporting Information

Additional supporting information can be found online in the Supporting Information section.

## Supporting information


**Supporting Information** Table S1: Ascertainment for major depression and anxiety cases in the UK Biobank study. Table S2: The numbers of participants with missing lifestyle information. Table S3: Baseline characteristics across child‐to‐adult body size categories. Table S4: Baseline characteristics of included and excluded participants. Table S5: Correlations among the exposure contrast, potential explanatory factors and mental health outcomes. Table S6: Internal consistency of PHQ‐9 and GAD‐7. Table S7: Exploratory mediation estimates and model fit indices. Figure S1: Flowchart for the selection of the study population from the UK Biobank study. Figure S2: BMI residual plots. Figure S3: Definition and proportions for body size categories from childhood to adulthood across sexes. Figure S4: Major depression (A,B) and anxiety risk (C,D) across child and adult body size groups separately. Figure S5: Hazard ratios and 95% CIs for major depression and anxiety associated with different child–adult body size categories by sex. Figure S6: Sensitivity analysis of associations of child–adult body size categories with incident depression and anxiety. Figure S7: Sensitivity analysis of the mediating role of common chronic diseases (including T2D, hypertension and CVD) and lifestyle scores in the relationship between the most disadvantaged child‐to‐adult body size group.

## Data Availability

The data from the UK Biobank are available upon application at www.ukbiobank.ac.uk/register-apply.
