# Supplementary material for: Child‐To‐Adult Body Size Change and Risk of Depression and Anxiety: A Large Prospective Cohort Study
Source: Depress Anxiety. 2026 Sep 3;2026:6154705. doi: 10.1155/da/6154705 (PMC13540993; doi:10.1155/da/6154705)
Supplement: Supplementary file 1 [file DA-2026-6154705-s001.docx]

**Child-to-adult body size change and risk of depression and anxiety: a large prospective cohort study**

[S](#_Toc18357)**[upplemental Methods](#_Toc18357)**	[2](#_Toc18357)

**[Table S1](#_Toc13393)** [Ascertainment for major depression and anxiety cases in the UK Biobank study	4](#_Toc13393)

**[Table S2](#_Toc16080)** [The numbers of participants with missing lifestyle information	5](#_Toc16080)

**[Table S3](#_Toc23628)** [Baseline characteristics across child to adult body size categories.	6](#_Toc23628)

**[Table S4](#_Toc799)** [Baseline characteristics of included and excluded participants.	7](#_Toc799)

**[Table S5](#_Toc11837)** [Correlations among the exposure contrast, potential explanatory factors, and mental health outcomes.	8](#_Toc11837)

**[Table S6](#_Toc13777)** [Internal consistency of PHQ-9 and GAD-7.	9](#_Toc13777)

**[Table S7](#_Toc3390)** [Exploratory mediation estimates and model fit indices.	10](#_Toc3390)

**[Figure S1](#_Toc25007)** [Flowchart for the selection of the study population from the UK Biobank study.	11](#_Toc25007)

**[Figure S2](#_Toc9375)** [BMI residual plots.	12](#_Toc9375)

**[Figure S3](#_Toc27061)** [Definition and proportions for body size categories from childhood to adulthood across sexes.	13](#_Toc27061)

**[Figure S4](#_Toc17589)** [Major depression (A-B) and anxiety risk (C-D) across child and adult body size groups separately.	14](#_Toc17589)

**[Figure S5](#_Toc31002)** [Hazard ratios and 95% CIs for major depression and anxiety associated with different child–adult body size categories by sex.	15](#_Toc31002)

**[Figure S6](#_Toc32554)** [Sensitivity analysis of associations of child–adult body size categories with incident depression and anxiety: censoring up to Dec 31, 2019.	16](#_Toc32554)

**[Figure S7](#_Toc26864)** [Sensitivity analysis of the mediating role of common chronic diseases (including T2D, hypertension, and CVD) and lifestyle scores in the relationship between the most disadvantaged child-to-adult body size group	17](#_Toc26864)

# Supplemental Methods

**Assessment of mediating variables**

**Lifestyles**: All information on lifestyles was self-reported using a touchscreen questionnaire at baseline (2006-2010) (https://biobank.ndph.ox.ac.uk/showcase/). We constructed an overall healthy lifestyle score based on six lifestyle factors (i.e., smoking status, alcohol intake, physical activity, sleep duration, sedentary time and diet). Details of assessment of health behaviors were shown as follows:

| **UK Biobank** | | | |
| --- | --- | --- | --- |
| **Lifestyle factor** | **Questionnaire** | **Healthy habit** | **Unhealthy habit** |
| Smoking status | "Do you smoke tobacco now?" and "In the past, how often have you smoked tobacco?" | past or never smoker | current |
| Alcohol intake | “About how often do you drink alcohol?” | ≤ 4 times week | Daily or almost daily |
| Physical activity | International Physical Activity Questionnaire short form – total time walking or moderate or vigorous-intensity physical activity | ≥150 min/week moderate or ≥ 75 min/week vigorous physical activity | <150 min/week moderate or < 75 min/week vigorous physical activity |
| Sleep duration | “About how many hours sleep do you get in every 24 hours?” | ≥7 or ≤9h/day | <7 or >9h/day |
| TV viewing / sedentary time | “In a typical day, how many hours do you spend watching TV?” | < 4 h/day | ≥ 4 h/day |
| Diet score (From Touchscreen questionnaire at baseline. Healthy diet was defined as meeting five or more ideal diet components) | | | |
| Fruit | "About how many of …. would you eat per day?” Separate questions for pieces of fresh and dried fruit. | ≥3 servings/day | < 3 servings/day |
| Vegetable intake | "About how many of …. would you eat per day?” Separate questions for tablespoons of salad or cooked/raw vegetables. | ≥3 servings/day | < 3 servings/day |
| Oily fish intake | "How often do you eat oily fish? (e.g. sardines, salmon, mackerel, herring)" | ≥2 servings/week | < 2 servings/week |
| Red meat intake | "How often do you eat…?” Separate questions for Beef / lamb or mutton / pork (excluding processed meats such as ham or bacon). | ≤1.5 servings/week | >1.5 servings/week |
| Processed meat intake | "How often do you eat processed meats (such as bacon, ham, sausages, meat pies, kebabs, burgers, chicken nuggets)?" | ≤1 servings/week | >1 servings/week |
| whole grains | "How many slices of bread do you eat each WEEK?" and "What type of bread do you mainly eat?"; "How many bowls of cereal do you eat a WEEK?" and  "What type of cereal do you mainly eat?" (brown bread, wholemeal and wholegrain bread, bran and oat cereal were defined as whole grains) | ≥3 servings/day | < 3servings/day |
| Refined grains | "How many slices of bread do you eat each WEEK?" and "What type of bread do you mainly eat?"; "How many bowls of cereal do you eat a WEEK?" and  "What type of cereal do you mainly eat?" (white and other type of bread, biscuit cereal, muesli and other type of cereal were defined as refined grains) | ≤1.5 servings/day | > 1.5 servings/day |

**Hypertension**: Hypertension was identified using cumulative hospital inpatient records. The definition was described according to the 9th and 10th revisions of the International Classification of Diseases (ICD-9 and ICD-10) and self-reported data fields with choice-, disease-, or procedure-specific codes.

| **Prevalent diseases** | **Self-reported** | **ICD-9** | **ICD-10** |
| --- | --- | --- | --- |
| Hypertension | 6150 (4), 20002 (1065,1072), 6153 (2), 6177 (2) | 401-405 | I10-I13, I15, O10 |

**Cardiovascular disease**: Cardiovascular disease was identified using cumulative hospital inpatient records. The definition was described according to the 9th and 10th revisions of the International Classification of Diseases (ICD-9 and ICD-10) and self-reported data fields with choice-, disease-, or procedure-specific codes.

| **Prevalent diseases** | **Self-reported** | **ICD-9** | **ICD-10** |
| --- | --- | --- | --- |
| Cardiovascular disease* | 6150 (1,2,3), 20002 (1074,1075,1081,1583,1086, 1491) | 410-414, 430-434, 436 | I20-I25, I60-I64 |

*Contains coronary heart disease and stroke.

**Type 2 Diabetes**: Patients with type 2 diabetes identified by using the algorithms method developed by the UK Biobank study.

| **Prevalent diseases** | **Field ID** |
| --- | --- |
| Diabetes | 2443, 4041, 2976, 6177, 6153, 2986, 20002, 20003, 20009 |

# Table S1 Ascertainment for major depression and anxiety cases in the UK Biobank study

|  | **ICD-10** |
| --- | --- |
| Depression | F32.0, F32.1, F32.2, F32.3, F32.8, F32.9, F33.0,  F33.1, F33.2, F33.3, F33.4, F33.8, F33.9 |
| Anxiety | F40.0, F40.1, F40.2, F40.8, F40.9, F41.0, F41.1, F41.2, F41.3, F41.8, F41.9, F42.0, F42.1, F42.2, F42.8, F42.9, F43.0, F43.1, F43.2, F43.8, F43.9 |

# Table S2 The numbers (percentages) of participants with missing lifestyle information.

|  | **Number** | **Percentage (%)** |
| --- | --- | --- |
| Lifestyle score | 158634 | 35.42 |
| Smoking status | 1394 | 0.31 |
| Alcohol intake | 219 | 0.05 |
| Physical activity | 81733 | 18.25 |
| Sleep duration | 2027 | 0.45 |
| TV viewing / sedentary time | 2783 | 0.62 |
| Diet | 100180 | 22.37 |

# Table S3 Baseline characteristics across child to adult body size categories.

|  | **Child_Low_** | **Child_Average_** | **Child_High_** | **Child_Low_** | **Child_Average_** | **Child_High_** | **Child_Low_** | **Child_Average_** | **Child_High_** | **Total** |
| --- | --- | --- | --- | --- | --- | --- | --- | --- | --- | --- |
| **Characteristic** | **Adult_Low_** | | | **Adult_Average_** | | | **Adult_High_** | | |  |
| **Number of participants** | 60134 | 74371 | 12551 | 70071 | 122455 | 36813 | 16851 | 32513 | 22062 | 447821 |
| **Age, mean (SD)** | 56.10 (8.08) | 56.81 (7.96) | 56.29 (7.67) | 57.17 (8.07) | 57.04 (8.07) | 56.25 (7.94) | 57.40 (7.91) | 56.90 (7.89) | 55.45 (7.86) | 56.73 (8.02) |
| **Male, No. (%)** | 31378 (52.18%) | 34584 (46.50%) | 5009  (39.91%) | 32709 (46.68%) | 56798 (46.38%) | 14947 (40.60%) | 6884  (40.85%) | 13072 (40.21%) | 8061  (36.54%) | 203442  (45.43%) |
| **Townsend deprivation index, mean (SD)** | -1.63 (2.92) | -1.64 (2.93) | -1.59 (2.97) | -1.48 (2.97) | -1.67 (2.85) | -1.49 (2.97) | -0.56 (3.29) | -1.06 (3.12) | -0.92 (3.15) | -1.49 (2.97) |
| **Educational attainment (university), No. (%)** | 24595 (40.90%) | 29065 (39.08%) | 5383  (42.89%) | 19297 (27.54%) | 38034 (31.06%) | 12301 (33.41%) | 3086  (18.31%) | 7790 (23.96%) | 6141 (27.84%) | 145692  (32.53%) |
| **Diabetes, No. (%)** | 976  (1.62%) | 977  (1.31%) | 157  (1.25%) | 3165  (4.52%) | 3916  (3.20%) | 1353  (3.68%) | 2368  (14.05%) | 3423 (10.53%) | 2640 (11.97%) | 18975  (4.24%) |
| **Hypertension, No. (%)** | 11288 (18.77%) | 12938 (17.40%) | 2207  (17.58%) | 22913 (32.70%) | 35162 (28.71%) | 10741 (29.18%) | 8862  (52.59%) | 15175 (46.67%) | 10421  (47.24%) | 129707  (28.96%) |
| **Cardiovascular disease, No. (%)** | 2713  (4.51%) | 3328  (4.47%) | 489  (3.90%) | 5475  (7.81%) | 7804 (6.37%) | 2276  (6.18%) | 2207  (13.10%) | 3123 (9.61%) | 2067  (9.37%) | 29482  (6.58%) |
| **Unhealthy lifestyle score, median (IQR)** | 2.00 (1.00-2.00) | 2.00 (1.00-2.00) | 2.00 (1.00-2.00) | 2.00 (1.00-3.00) | 2.00 (1.00-2.00) | 2.00 (1.00-2.00) | 2.00 (1.00-3.00) | 2.00 (1.00-3.00) | 2.00 (1.00-3.00) | 2.00  (1.00-2.00) |

**Table S4** Baseline characteristics of included and excluded participants.

| **Characteristic** | **Included (n=447,821)** | **Excluded (n=54,580)** | **SMD** |
| --- | --- | --- | --- |
| Age, years | 56.73 (8.02) | 54.85 (8.52) | 0.228 |
| Male sex | 203,442 (45.4) | 25,639 (47.0) | -0.031 |
| White ethnicity | 447,821 (100.0) | 24,782 (45.4) | 1.551 |
| College/university degree | 145,692 (32.5) | 15,429 (30.9) | 0.035 |
| Townsend deprivation index | -1.49 (2.97) | 0.34 (3.59) | -0.556 |
| BMI, kg/m2 | 27.38 (4.76) | 27.93 (5.17) | -0.112 |
| Childhood body size: Child_Low_ | 147,056 (32.8) | 16,562 (30.9) | 0.043 |
| Childhood body size: Child_Average_ | 229,339 (51.2) | 20,242 (37.7) | 0.274 |
| Childhood body size: Child_High_ | 71,426 (15.9) | 6,726 (12.5) | 0.098 |
| Lifestyle score | 1.81 (1.05) | 1.90 (1.10) | -0.079 |
| Type 2 diabetes | 18,975 (4.2) | 4,988 (9.1) | -0.197 |
| Hypertension | 129,707 (29.0) | 18,765 (34.4) | -0.117 |
| Cardiovascular disease | 29,482 (6.6) | 5,206 (9.5) | -0.109 |

*SMD, standardized mean difference*

**Table S5** Correlations among the exposure contrast, potential explanatory factors, and mental health outcomes.

| **Variable_1** | **Variable_2** | **N** | **Spearman_r** | **P_value** |
| --- | --- | --- | --- | --- |
| Child_Low_-Adult_High_ contrast | Lifestyle score | 89982 | 0.097 | <0.001 |
| Child_Low_-Adult_High_ contrast | CVD | 139306 | 0.085 | <0.001 |
| Child_Low_-Adult_High_ contrast | T2D | 139306 | 0.171 | <0.001 |
| Child_Low_-Adult_High_ contrast | Hypertension | 139306 | 0.167 | <0.001 |
| Lifestyle score | Depression | 89982 | 0.045 | <0.001 |
| CVD | Depression | 139306 | 0.037 | <0.001 |
| T2D | Depression | 139306 | 0.03 | <0.001 |
| Hypertension | Depression | 139306 | 0.04 | <0.001 |
| Lifestyle score | Anxiety | 89982 | 0.025 | <0.001 |
| CVD | Anxiety | 139306 | 0.024 | <0.001 |
| T2D | Anxiety | 139306 | 0.012 | <0.001 |
| Hypertension | Anxiety | 139306 | 0.033 | <0.001 |

*The exposure contrast compared the child_Low_-adult_High_ group with the child_Average_ - adult_Average_ group. Correlations are Spearman rank correlations.*

**Table S6** Internal consistency of PHQ-9 and GAD-7.

| **Scale** | **Complete N** | **Items** | **Cronbach’alpha** |
| --- | --- | --- | --- |
| PHQ-9 | 144475 | 9 | 0.842 |
| GAD-7 | 145107 | 7 | 0.898 |

**Table S7** Exploratory mediation estimates and model fit indices.

| **Outcome** | **Mediator** | **Indirect** | **Direct** | **Total** | **Mediated %** | **Med. R2** | **Med. AIC** | **R^2^** | **AIC** |
| --- | --- | --- | --- | --- | --- | --- | --- | --- | --- |
| Depression | Lifestyle score | 0.0027 | 0.0268 | 0.0296 | 9.3 | 0.0417 | 1297097.3 | 0.0511 | 168833.6 |
| Depression | CVD | 0.0004 | 0.0267 | 0.0271 | 1.6 | 0.1851 | 177244.3 | 0.0511 | 168833.6 |
| Depression | T2D | 0.0008 | 0.0267 | 0.0276 | 3.0 | 0.1867 | 127852.9 | 0.0511 | 168833.6 |
| Depression | Hypertension | 0.0023 | 0.0271 | 0.0294 | 7.9 | 0.1458 | 460527.4 | 0.0511 | 168833.6 |
| Anxiety | Lifestyle score | 0.0015 | 0.0151 | 0.0166 | 9.1 | 0.0417 | 1297097.3 | 0.0395 | 151976.2 |
| Anxiety | CVD | 0.0003 | 0.015 | 0.0153 | 1.7 | 0.1851 | 177244.3 | 0.0395 | 151976.2 |
| Anxiety | T2D | 0.0 | 0.015 | 0.015 | 0.3 | 0.1867 | 127852.9 | 0.0395 | 151976.2 |
| Anxiety | Hypertension | 0.002 | 0.0152 | 0.0172 | 11.4 | 0.1458 | 460527.4 | 0.0395 | 151976.2 |

*Effects are reported on the risk-difference scale for the childLow-adultHigh group compared with the childAverage-adultAverage group. R2 for logistic models is McFadden's pseudo-R2; R2 for the linear mediator model is ordinary R2.*

# Figure S1 Flowchart for the selection of the study population from the UK Biobank study.

# Figure S2 BMI residual plots.

# Figure S3 Definition and proportions for body size categories from childhood to adulthood across sexes.

# Figure S4 Major depression (A-B) and anxiety risk (C-D) across child and adult body size groups separately.

Model was adjusted for age, sex, educational attainment, Townsend deprivation index, and assessment centers. Child and adult body size being mutually adjusted in analyses.

# Figure S5 Hazard ratios and 95% CIs for major depression and anxiety associated with different child–adult body size categories by sex.

Model was adjusted for age, sex, educational attainment, Townsend deprivation index and assessment centers.

# Figure S6 Sensitivity analysis of associations of child–adult body size categories with incident depression and anxiety: censoring up to Dec 31, 2019.

Model was adjusted for age, sex, educational attainment, Townsend deprivation index and assessment centers.

# Figure S7 Sensitivity analysis of the mediating role of common chronic diseases (including T2D, hypertension, and CVD) and lifestyle scores in the relationship between the most disadvantaged child-to-adult body size group (the childLow-adultHigh group) and (A) depression and (B) anxiety: using participants with complete lifestyle information.

ADE, average direct effects; ACME, average causal mediation effects.
